# Rapid Screening Evaluation of SARS-CoV-2 IgG Assays Using Z-Scores to Standardize Results

**DOI:** 10.3201/eid2610.202632

**Published:** 2020-10

**Authors:** Marie K. Das, Anu Chaudhary, Andrew Bryan, Mark H. Wener, Susan L. Fink, Chihiro Morishima

**Affiliations:** University of Washington, Seattle, Washington, USA

**Keywords:** severe acute respiratory syndrome coronavirus 2, SARS-CoV-2, coronavirus, viruses, coronavirus disease, COVID19, antibody, serologic analysis, IgG, screening, qualitative results, quantitative results, performance, standardization, z-scores, respiratory infections, zoonoses

## Abstract

Many serologic tests are now available for measuring severe acute respiratory syndrome coronavirus 2 antibodies to evaluate potential protective immunity and for seroprevalence studies. We describe an approach to standardizing positivity thresholds and quantitative values for different assays that uses z-scores to enable rapid and efficient comparison of serologic test performance.

Measurement of severe acute respiratory syndrome coronavirus 2 (SARS-CoV-2) antibodies has become increasingly important for assessing potential immunity as the coronavirus disease (COVID-19) pandemic evolves. Most immunoassays for SARS-CoV-2 antibodies yield quantitative converted to qualitative results, requiring a positivity threshold whose basis might be unclear when provided by the manufacturer. Using specimens from hospitalized patients with acute COVID-19 and archived pre–COVID-19 serum samples, we established standardized positivity thresholds and quantitative values for multiple commercially available immunoassays, which enabled efficient screening comparison of serologic reagents.

Remnant blood specimens were selected from a convenience sample of patients given diagnoses of COVID-19 by using a laboratory-developed reverse transcription PCR (*1*). Serologic testing was performed at the University of Washington Clinical Immunology Laboratory after institutional review board approval (study #9954).

We used 4 commercial SARS-CoV-2 IgG ELISA kits: Euroimmun IgG Kit (lot no. E200225BV; https://www.euroimmun.com) with recombinant structural protein (spike [S] 1 domain) as target (*2*); Epitope Diagnostics (EPI) EDI Novel Coronavirus COVID19 IgG Kit (lot no. P529, http://www.epitopediagnostics.com) with nucleocapsid protein (NP) as target; ImmunoDiagnostics anti-SARS-CoV-2-NP IgG Kit (lot no. N0313; https://www.immunodiagnostics.com.hk) with NP as target; and ImmunoDiagnostics anti-SARS-CoV-2-S1RBD IgG Kit (lot no. S0313) with receptor-binding domain (RBD) of the S1 protein as target. All testing was performed according to manufacturer’s protocols.

To standardize results, optical density (OD) scores for each sample were converted to z-scores by using the equation z-score = (test OD – mean negative control OD)/mean negative control SD. For Euroimmun, the OD ratio was calculated by using a kit calibrator. Negative control serum samples had been collected during April 2015–November 2019 from 25 healthy community blood donors. A conservative z-score >3 (number of SDs above the negative control mean) was considered positive to minimize false-positive results.

A total of 23 samples were tested from a cohort of 11 patients with reverse transcription PCR–confirmed COVID-19. The standardized results illustrate the differing sensitivities of the 4 assays (Table). As expected, positive results were strongly associated with time after symptom onset, consistent with results of previous studies (*2*–*5*). In contrast to the other assays, the ImmunoDiagnostics S1RBD Kit did not show typical seroconversion, although an assay that used RBD from a local academic laboratory demonstrated seroconversion (data not shown).

**Table:** Results from 4 immunoassays for severe acute respiratory syndrome coronavirus 2 IgG using a standardized z-score threshold of 3*

Days from symptom onset	EU IgG	EPI IgG	ID NP IgG	ID S1RBD IgG
0–6	0/4 (0)	0/4 (0)	2/4 (50)	2/4 (50)
7–13	1/11 (9)	7/11 (64)	7/11 (64)	1/11 (9)
14–20	7/8 (88)	8/8 (100)	8/8 (100)	0/8 (0)

*Values are no. positive/no. tested (%). EU, Euroimmun (https://www.euroimmun.com); EPI, Epitope Diagnostics (http://www.epitopediagnostics.com); ID, ImmunoDiagnostics (https://www.immunodiagnostics.com.hk); NP, nucleocapsid protein; RBD, receptor-binding domain; S1, spike protein.

We provide serial results for 3 patients over the first 3 weeks after symptom onset (Figure). Using the z-score threshold ≥3, we found that patient 1, who recovered, had positive IgG responses by 3 assays. Patient 10 had IgG responses detected by 2 assays, and patient 11 had IgG responses detected by 3 assays (Figure, panel A); both of these patients died. Antibody responses measured by different kits standardized as z-scores showed relative differences from raw OD results (Figure, panel B). A definitive comparison between quantitative values would require further characterization and optimization of quantitative performance. However, we show the benefit of comparing results from different assays in a standardized way (Figure). Although our small sample size precludes any conclusions regarding seroconversion and relationship to disease course, variability in antibody response kinetics between persons was demonstrated.

**Figure:**
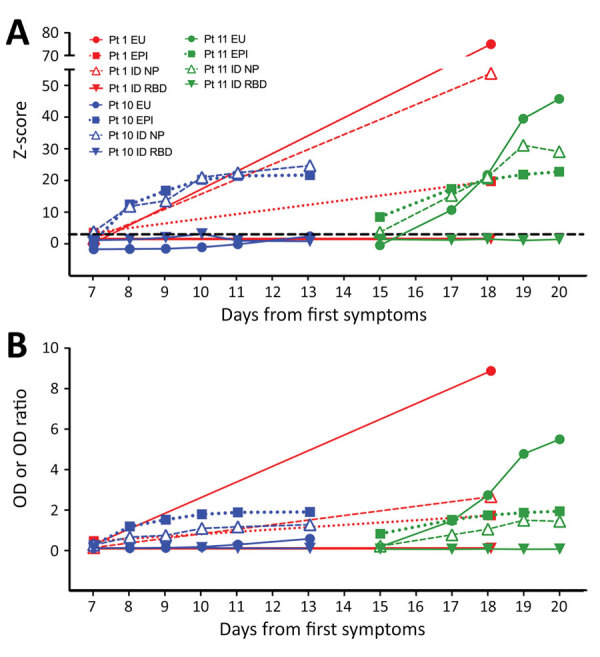
Results from 4 severe acute respiratory syndrome coronavirus 2 IgG assays, by days from first symptoms, for 3 patients with serial results demonstrating seroconversion. Immunoassay results are shown as z-scores (A), calculated from OD or OD ratio (EU) results (B) as described, and respective negative control population means and SDs for each assay (n = 25). Control samples were collected from healthy persons during 2015–2019 and tested with all 4 assays. For all patients, results from different assays are indicated as EU IgG (solid circles); EPI IgG (solid squares); ID NP IgG (open triangles); and ID S1RBD IgG (solid triangles). Red indicates results for patient 1, blue indicates results for patient 10, and green indicates results for patient 11. Dashed line in panel A indicates the z-score positivity threshold of 3. EPI, Epitope Diagnostics (http://www.epitopediagnostics.com); EU, Euroimmun (https://www.euroimmun.com); ID, ImmunoDiagnostics (https://www.immunodiagnostics.com.hk); NP, nucleocapsid protein; OD, optical density; RBD, receptor-binding domain; S, spike protein.

Among 25 negative control samples, 6 were positive by EPI-provided thresholds, but negative by the other tests, suggesting that the recommended EPI cutoff was inappropriately low. All 25 control results were included in EPI z-score calculations, and led to a positivity threshold higher than recommended by EPI. In contrast, our local population-based z-score cutoff was lower than the threshold recommended by EU. Despite these differences, qualitative results obtained by using manufacturer-supplied cutoffs and z-scores were identical for EU and EPI results for our limited sample set. The ID kits did not include a recommended positivity threshold, but use of a z-score of 3, and results generated by using the same local negative control samples as the other kits facilitated an unbiased comparison.

Three patients had discordant qualitative results for Euroimmun, EPI, and ImmunoDiagnostics NP assays. Patient 10 had nucleocapsid responses (EPI and ImmunoDiagnostics NP) but no S1 response (Euroimmun) detected, and patients 4 and 5 had nucleocapsid antibody responses just above positivity thresholds detected by 1 but not the other assays. Different studies have reported serologic results using in-house (*2*) or manufacturer-recommended thresholds (*6**,**7*). The choice of thresholds could affect identification of immune versus nonimmune persons and of seroprevalence in a population, particularly if asymptomatic or mildly affected persons have low levels of antibodies.

Clinical assay validation is always required, but is particularly needed for COVID-19 antibody assays given the current emergency use climate with limited regulatory oversight. Use of pre–COVID-19–era reference specimens to calculate standardized z-score results for immunoassays with different or no manufacturer-recommended cutoffs, and a small sample of locally collected specimens from SARS-CoV-2–infected persons enabled rapid comparison. As attention turns to calculated measurement of vaccine-induced responses, comparison of quantitative assays is likely to become important, and z-scores (with >20 control samples tested once) should also find utility in that setting. Finally, careful evaluation of manufacturer-recommended positivity thresholds for SARS-CoV-2 qualitative antibody tests is warranted.
